# The Dental News

**Published:** 1898-04

**Authors:** 


### The Dental News.

A monthly journal devoted to the interests of the profession and the advancement of dental science. Edited by Dr. E. Weber, M.D., D.D.S., Washington, D. C.

This journal began its issues in August, 1897, and has made its appearance from month to month to the present time. It is an unpretentious publication of some sixteen pages, filled with interesting matter of interest to all, and especially the busy man who may not have time and opportunity to read and digest the longer aaticles that are found in the larger journals.

The profession at Washington and vicinity certainly needs a vehicle such as this for thought and experience, and we predict that in the near future it will be found necessary to add to its pages to answer the demand that may be made upon it.

